# Esophageal adenocarcinoma with leukemoid reaction: a case report

**DOI:** 10.1186/s13019-019-0893-z

**Published:** 2019-04-08

**Authors:** Ge Yu, Huaijun Ji, Chuizheng Meng, Yixuan Huang, Guogang Gao, Chuanping Liu, Shanlei Wang, Lei Zhang, Jin Ju

**Affiliations:** Department of Thoracic Surgery, Weihai Municipal Hospital, 70 Heping Road, Weihai, 264200 Shandong China

**Keywords:** Leukemoid reaction (LR), Hyperleukocytosis, Esophageal adenocarcinoma, Esophagectomy

## Abstract

**Background:**

Leukemoid reaction (LR) is defined as a reactive leucocytosis with WBC counts exceeding 50,000/mm^3^, and a significant increase in early neutrophil precursors. LR may be a paraneoplastic manifestation of various malignant tumors. Tumor-related LR is a kind of neoplastic syndrome, unrelated to an infection or other diseases.

**Case presentation:**

A 74-year-old male visited a local doctor with a 20-day history of progressive dysphagia. The complete blood count revealed leucocytosis. Bone marrow aspirates and a biopsy confirmed LR and excluded chronic myelogenous leukemia. Following radical esophagectomy for an adenocarcinoma the WBC counts successively decreased to 10,450/mm^3^ and 8670/mm^3^ within 1 week and 1 month, respectively.

**Conclusion:**

We report a rare case of esophageal adenocarcinoma complicated with *excessive leucocytosis* caused by paraneoplastic LR; we also present a review *of literature* and an investigation of the clinical features. To our knowledge, this is the first report of LR associated with esophageal adenocarcinoma.

## Background

A leukemoid reaction (LR) is defined as white blood cell (WBC) count exceeding 50,000/mm^3^, owing to extramedullary causes [1]. LR is most often caused by serious infection [2], but can also be a paraneoplastic manifestation of several cancers, including lung, gastrointestinal, genitourinary, ovarian, and head and neck cancers, and hepatocellular carcinoma [3]. Hughes and Charles first reported a case of LR resulting from a carcinoma of the suprarenal gland in 1952 [4]. Since then, great efforts have been made to elucidate the mechanism of LR. Paraneoplastic syndromes, such as tumour fever and LR, have been described in literature [5, 6]. However, studies on the incidence and course of LR in solid tumours are scarce [7]. One study reported that the occurrence of LR in solid tumours is approximately 1–4% [8]. However, owing to few accounts in medical literature, the causes of this occurrence are not well understood. Here, we report a rare case of esophageal adenocarcinoma complicated with hyperleukocytosis caused by a paraneoplastic leukemoid reaction (PLR).

## Case presentation

**Fig. 1:**
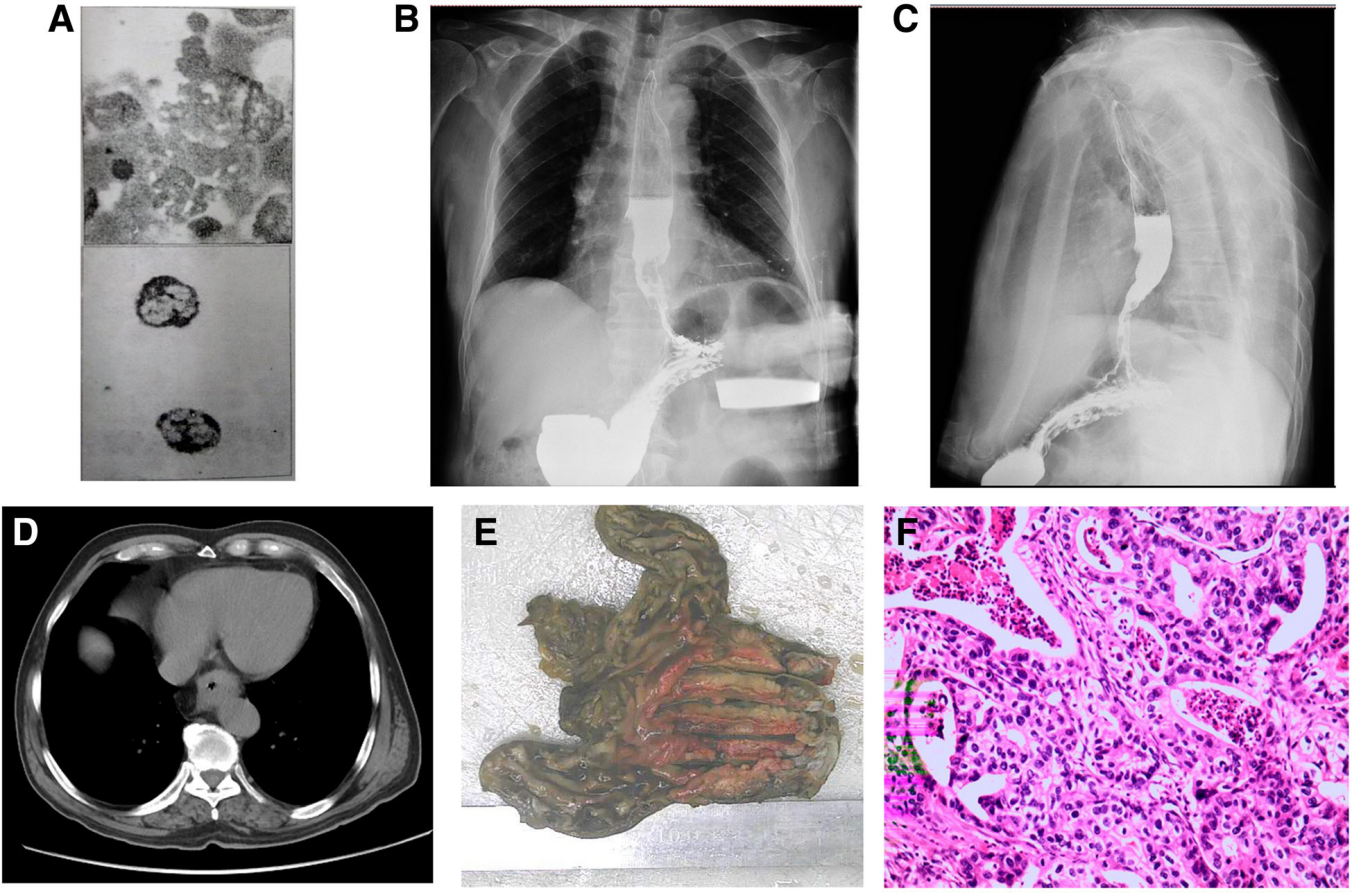
Test results of a patient with paraneoplastic leukemoid reaction associated with esophageal adenocarcinoma. **a** Bone marrow aspirate and biopsy showing granulocytosis which had proliferated actively, abundant droplet bodies in the cytoplasm, and increased megakaryocytes. **b** Anteroposterior radiograph of the upper gastrointestinal tract showing amass in the lower esophagus. **c** Lateral cephalometric radiograph of the upper gastrointestinal tract showing the lower esophageal mass. **d** Chest computed tomography (CT) showing thickening of the esophageal wall with corresponding regions of luminal stenosis. **e** The resected specimen showing an elevated tumour measuring 6.5 × 6.0 × 1 cm in the lower segment of the esophagus and gastroesophageal junction. **f** Microscopy, showing a moderately differentiated adenocarcinoma invading the serous layer

A 74-year-old male visited a local doctor in May 2018 due to a 20-day history of progressive dysphagia. He had no other symptoms suggestive of esophageal cancer. Electronic gastroscopy showed an elevated tumour in the lower segment of his esophagus, which revealed an esophageal adenocarcinoma on biopsy. The complete blood count revealed a total leucocyte count of 24,870/mm^3^ and the peripheral blood smear showed differential counts of 89, 5, and 4% for neutrophils, lymphocytes, and monocytes 4%, respectively. Since he had no fever, the treating physician did not suspect an inflammatory reaction or abscess, but considered this to be a manifestation of a secondary haematologica disorder. Chest computed tomography (CT) showed thickening of the wall of the esophagus, pulmonary inflammation, and mild bronchiectasis. Aspirates and a biopsy from the bone marrow (Fig. 1a) revealed granulocytosis that had proliferated actively, abundant droplet bodies in the cytoplasm, and an increased number of megakaryocytes. The positivity rate of alkaline phosphatase expression was also increased. These results confirmed a diagnosis of LR and excluded chronic myelogenous leukaemia (CML). The patient was referred to our hospital in Jun 2018 for further evaluation and treatment. The patient had an unremarkable family history. On examination, there were no palpable supraclavicular and bilateral cervical lymph nodes, and his body temperature was normal. His vitals were stable with a normal blood pressure, regular heart rate, and normal pulse rate. However, his weight had decreased by 4.5 kg since he had begun to experienc dysphagia. Laboratory investigations included a routine blood test (total WBC: 29,960/mm3, neutrophils: 89.5%, lymphocytes: 7.5%, monocytes: 2.3%, and haemoglobin: 138 g/l) and analysis of tumour markers (alpha-fetoprotein: 1.41 ng/ml, squamous cell carcinoma: 0.1 ng/ml, and carcinoembryonic antigen: 1.01 ng/ml). C-reactive protein was elevated (14.36 mg/dl). Yhe uric acid levels and liver and kidney function tests were within normal limits.Hyperleukocytosis, elevated C-reactive protein, and the neurologic features on the day of admission were indicative of an infectious pathology. Initially, the hyperleukocytosis was considered to be related to an infection. Subsequently, the patient was treated with biapenem (0.3 g twice dailyfor days 1–6) and voriconazole (200 mg twice daily for days 1–6). His WBC counts were 25,290/mm^3^ and 28,730/mm^3^ on the 4th and 6th days of treatment, respectively. All the blood cultures taken were negative. Persistent leucocytosis after 6 days of antibiotic treatment indicated that this could not be caused solely by infection. We therefore concluded that the LR was most probably caused by esophageal adenocarcinoma. A radiographic examination of the upper gastrointestinal tract (Fig. 1b-c) revealed a mass in the lower esophagus. A chest CT (Fig. 1d) showed thickening of the wall of the esophagus, corresponding regions of luminal stenosis, and several enlarged lymph nodes around the lesser curvature of the esophagus. No primary or metastatic tumours were observed. An abdominal ultrasound was performed, which identified no metastases in the liver. A radical esophagectomy was then performed. The resected specimen showed an elevated tumour measuring 6.5 × 6.0 × 1 cm in the lower segment of the esophagus and gastroesophageal junction (Fig. 1e). Microscopically, the tumour was a moderately differentiated adenocarcinoma invading the serous layer (Fig. 1f). A prominent intravascular proliferation of tumour cells was observed, which occasionally formed tumour thrombi. Clearance of lymph nodes during the radical esophagectomy revealed no grossly enlarged nodes. The tumour was diagnosed to be of stage IIB (T3N0M0), according to the American Joint Committee on Cancer guidelines.

Postoperatively, the WBC counts successively decreased to 10,450/mm^3^ within 1 week and 8670/mm^3^ at 1 month. The patient received 4 cycles of nedaplatin (200 mg on day 1), fluorouracil (1.0 g on days 1--5), and calcium folinate (0.3 g on days 1--5), and has been free of recurrence for 6 months.

## Discussion and conclusion

A WBC count exceeding 50,000/mm^3^ associated with extramedullary causes is termed a LR. It results in a severe left shift with the presence of immature myeloid cells in the peripheral blood [1]. Experts now agree that it is not essential the WBC count exceeds 50,000/mm^3^ to be diagnose LR; counts beyond the normal upper limit (4000-10,000/mm^3^) are sufficient [1]. Some of these cases have been misdiagnosed as acute leukaemia due to the severe elevation in the WBC count. However, leukaemia commonly presents with anaemia and immature myeloid cells on a peripheral blood smear, and bone marrow biopsies typically show hypercellularity. [2, 9] The evidence is available directly by bone marrow biopsy and by an inverse correlation between tumour response and leucocytosis, as well as an increased PLR [10]. LR typically presents with elevated levels of mature polymorphonuclear cells and immature cells of granulocyte lineage on peripheral smears. Leucocyte alkaline phosphatase is typically normal or elevated in LR. Leucocytosis is a typical response to infections, drugs, chromosomal abnormalities, and paraneoplastic phenomen [11]. LR can also be a paraneoplastic manifestation of several cancers [3]. In solid tumours, LR can be caused by an increased granulocyte colony stimulating factor or other growth factors including granulocyte-macrophage colony-stimulating factor (GM-CSF) and interleukin-6 (IL-6), which is thought to be produced by malignant cells of the cervix, pancreas, hepatocellular carcinoma, and lung cancer [3]. In the normal physiologic state, GM-CSF can stimulate proliferation and differentiation of neutrophil colony-forming cells. GM-CSF can also alter several functions of mature neutrophils [12]. During LR, over-expression of GM-CSF is associated with subsequent leucocytosis [13]. Mari K et al. reported a patient with esophageal squamous cell carcinoma (ESCC) with remarkable leucocytosis and high G-CSF levels [14]. ESCC is a disease with a poor prognosis, and the prognosis of ESCC with LR is considered even poorer [14]. Shun et al. also reported a case of aggressive G-CSF producing ESCC with increased WBC, and neutrophil counts, and increased C-reactive protein(CRP) [15]. The counts of WBC and neutrophils, and serum CRP levels may be markers of its progression. The neutrophil counts are especially important as a marker of progression [15]. Antoine et al. reported that leucocytosis and neutrophilia were independent prognostic factors of poor overall survival (OS), progression free survival (PFS), and locoregional control (LRC) in patients with locally advanced ESCC undergoing exclusive chemoradiation [16]. Animal experiments have demonstrated that IL-6 can sustain neutrophil/macrophage colonies in vivo, and IL-6 can act on other immune cells, which may induce the production of several CSFs by the bone marrow [17]. Due to the rarity of PLR, studies on this disease have been very limited. HurtadoCordovi J et al. reported that only 77 (10%) among 758 patients who had solid tumours with hyperleukocytosis in their cohort had PLR [18]. The prognosis of patients with PLR is poor. According to a review, 78% patients died within 12 weeks of the first detection of hyperleukocytosis; only 10% of patients were observed to survive more than 1 year after successful treatment of the malignancy. Owing to the poor prognosis of patients with PLR, it is essential to rule out life-threatening causes prior to making a diagnosis of PLR. In terms of early diagnosis and subsequent disease management, staining for GM-CSF early in the course of the disease may provide valuable information. Treatment strategies are limited in patients with PLR, and these are rarely reported in the literature. The effective treatment for leucocytosis is removal of the inciting tumour. Unfortunately, extirpation is not always definitive [19]. In a retrospective, single-institution study, Granger and Kontoyiannis reported that patients with PLR who survived longer than 1 year had received effective antineoplastic therapy, chemotherapy, or surgery [20]. Moreover, it has been reported that 78% of patients either died or were discharged to hospice within 12 weeks of their initial extreme leucocyte count findings [21]. Here, we report a case of esophageal adenocarcinoma with an abnormal haematological picture dominated by LR. Our patient, underwent radical esophagectomy. Postoperatively, his WBC counts decreased to normal values, and he has been free of recurrence for 6 months.

In conclusion, an accurate and timely diagnosis of LR associated with esophageal adenocarcinoma and appropriate treatment strategies can improve the prognosis of patients with this rare condition.

## Abbreviations

CML: Chronic myelogenous leukemia
CRP: C-reactive protein
CT: Computed tomography
ESCC: Esophageal squamous cell carcinoma
GM-CSF: Granulocyte-macrophage colony-stimulating factor
HCC: Hepatocellular carcinoma
IL-6: Interleukin-6
LR: Leukemoid reaction
LR: Locoregional control
OS: Overall survival
PFS: Progression free survival
PLR: Paraneoplastic Leukemoid reaction
WBC: White blood cell count
